# An Algorithm for Critical Nodes Problem in Social Networks Based on Owen Value

**DOI:** 10.1155/2014/414717

**Published:** 2014-04-10

**Authors:** Xue-Guang Wang

**Affiliations:** ^1^Department of Computer Science, East China University of Political Science and Law, Shanghai 201620, China; ^2^Department of Computer Science, University College London (UCL), London WC1E 6BT, UK

## Abstract

Discovering critical nodes in social networks has many important applications. For finding out the critical nodes and considering the widespread community structure in social networks, we obtain each node's marginal contribution by Owen value. And then we can give a method for the solution of the critical node problem. We validate the feasibility and effectiveness of our method on two synthetic datasets and six real datasets. At the same time, the result obtained by using our method to analyze the terrorist network is in line with the actual situation.

## 1. Introduction

It is a basic process that happened in the network for the spread, diffusion, and cascade behavior of information. Considering that we plan to introduce new products, we can use the network feature which is called “word-of-mouth” or “viral marketing.” That is, we may find out some individuals with influence and let them recommend the product to their friends so that such a cascade spreads by the greatest extent in the people. How to choose these influential individuals is called critical node problem (CNP). An effective solution for the problem has an important practical value [1]. For example, we can find out the leaders quickly in the criminal relationship network; in the power network, we can protect important circuit breakers and power units for effectively preventing large-scale blackouts caused by cascading failure; in the disease network, we can specifically isolate the source of the disease and block its spread and diffusion; we can discover the initiators and avoid the butterfly effect in the rumor network.

This paper gives a solution for the CNP, which assigns a marginal contribution for every node in a community of social networks using the solution concept and union concept of cooperative games. Then we sort all nodes by their contribution and obtain critical nodes according to some rules. The rest of the paper is organized as follows.  Section 2 introduces two basic diffusion models and related background knowledge. An algorithm based on Owen value is presented in Section 3 and validated in Section 4. We conclude the paper in Section 5.

## 2. Backgrounds

### 2.1. Diffusion Models

The models for information propagation on networks have been widely studied [2–4]. We consider two basic models in this paper: independent cascade model (ICM) and linear threshold model (LTM) [5]. Some necessary definitions and hypotheses are firstly given.

A network is modeled as a graph *G* = (*V*, *E*) with vertices in *V* modeling the individuals in the network and edges in *E* modeling the relationship between individuals, where |*V* | = *n* and |*E* | = *m*. A vertex has two states: active and inactive, which means whether a product or idea is accepted by individuals or not. Assume that a vertex can only change from inactive to active and not vice versa; an inactive vertex can be activated by its active neighbor vertices and an active vertex can activate its inactive neighbors; the increment of activated vertices represents the dissemination of information.

#### 2.1.1. ICM

In this model, a propagation probability *p*_*u*,*v*_ is given for each edge (*u*, *v*) ∈ *E*; that is, vertex *v* is activated with probability *p*_*u*,*v*_ by *u*. When an initial set *A*_0_ of active vertices is given, the diffusion process spreads up according to the following randomized rule. When a vertex *u* is activated at time-step *i*, it has a single chance for activating its neighbor *v* with *p*_*u*,*v*_. If *u* succeeds, *v* will become active at time-step *i* + 1. Here, if *v* has multiple parent vertices that become active at time-step *i* for the first time, then their activation attempts are sequenced in an arbitrary order. Whether or not *u* succeeds, it cannot make any further attempts to activate *v* in subsequent steps. The process runs until no more activation is possible.

#### 2.1.2. LTM

In this model, vertex *v* is influenced by each neighbor *u* according to a weight *w*_*v*,*u*_ such that ∑_*v*_ *w*_*v*,*u*_ ≤ 1. Each vertex has a predefined threshold *θ*_*v*_ ∈ [0,1], which is chosen uniformly at random. When an initial set *A*_0_ with active vertices is given, the diffusion process unfolds according to the following randomized rule. All activated vertices at time-step *i* still keep active at time-step *i* + 1. Whether or not any inactivated vertex is activated is determined by its neighbors' weights such that ∑_*v*_ *w*_*v*,*u*_ ≥ *θ*_*v*_. The process runs until no more activation is possible.

The difference between ICM and LTM is that each attempt of activation is independent of the attempts by all the other active individuals while in the later model each inactive individual is influenced by the aggregated weight of all its active neighbors.

### 2.2. Problem Description

Given *G* = (*V*, *E*), influence spread model *M*, and a positive integer *k*, the critical node problem (CNP) is to find *k* vertices which maximize the extent of spread initiated by these vertices under the current model *M*. That is,

(1) $$\begin{array}{c} A=\underset{S\subseteq V,\left|S\right|\leq k}{\text{argmax}}\sigma \left(S\right), \end{array}$$

where *σ*(*A*) denotes the expected size of the set of vertices activated by an initial set *A*; *S* denotes any set with *k* vertices in *G*.

### 2.3. Related Works

Many statistical properties for social network analysis have been presented in the complex network theory, such as degree, clustering coefficient, and betweenness [6]. PageRank algorithm [3] and HITS algorithm [4] use the eigenvector centrality for ranking web pages; White and Smyth compute the node's relative importance by Markov centrality [7]; Shetty and Adibi discover the important nodes from the email network through graph entropy [5]; Li et al. identify influential Bloggers by artificial neural network [8]; Li et al. find out the Effectors by tree structure based on direct graph and dynamic linear programming [9]. Some scholars mine critical nodes for social networks based on specific network information [10–15].

Domingos and Richardson firstly studied the CNP as an algorithmic problem [16, 17]. Kempe et al. formulated the problem as the discrete optimization problem, proved that the problem is an NP-hard, and presented a greedy approximation algorithm (see Algorithm 1) which approximates the optimum within a factor of (1 − 1/*e*) [18, 19].

There is a key problem how to compute the value of *σ*(*A*) in Algorithm 1. Currently, we have not any efficient method to get its exact solution. However, we can use Monte-Carlo method to simulate the process of influence spread for obtaining approximate results by high probability. Assuming that every vertex run the process of *σ*(*A* ∪ {*v*})*R* times, and computing time of *σ*(*A*) is *O*(*m*), then the overall complexity for this greedy algorithm is *O*(*knRm*).

However, the method's efficiency severely restricts its scalability. Leskovec et al. proposed an optimized method referred to as the cost-effective lazy forward (CELF), which can speed up the above greedy algorithm [20]. Chen et al. did not improve the greedy algorithm itself but focused on computing mutual influence between the individuals in local network structure. They present a degree discount algorithm for ICM, which achieves almost matching influence thread with the greedy algorithm and is less than one-millionth of time of the greedy algorithm [21]. After that, they design a maximum influence arborescence model for the general ICM by restricting computations on the local influence regions of nodes [22] and a local directed acyclic graph algorithm for the general LTM [23] to solve the CNP. They also study the CNP in social networks when negative opinions may emerge and propagate [23].

## 3. A New Algorithm Based on Owen Value

### 3.1. Cooperative Game and Owen Value

Given a finite set of players *N*, cooperative game with transferable utility is a pair (*N*, *v*), characteristic function *v* : 2^*N*^ → ℝ, and *v*(*⌀*) = 0. For ∀*i* ∈ *N*, if payoff vector satisfies *x*_*i*_ ≥ *v*({*i*}) and ∑_*i*=1_^*N*^ *x*_*i*_ = *v*(*N*), then it is called an allocation of (*N*, *v*). The solution of cooperative game is a kind of allocation rule and the allocated payoff for every player denotes a method to measure the negotiation strength of the players in the game. Shapely presented a solution concept which finds out the only allocation distribution scheme from the solutions with different property; that is, it assigns the player's payoff according to the importance of every player for the game [24]. The Shapely value of the player *i* in the game (*N*, *v*) is

(2) Shi(v) =∑{S⊆N ∣ i∈S}(n−s)!(s−1)!n!(v(S∪{i})−v(S)), ∀i∈N,

where *n* = |*N*| and *s* = |*S*|.

However, the Shapely value does not consider the impact of coalition structure and Owen extends it [25]. Each union obtains its payoff from the game between the unions, and then the payoff is allocated by the internal game among the members of the union. All the payoffs are computed by the Shapely value.

Assume that *N* = {1,2,…, *n*} and *M* = {1,…, *m*}; a partition *P* = {*N*_1_, *N*_2_,…, *N*_*m*_} is a coalition structure on *N*. Let *N*_*k*_ be a union and  ⋃_1≤*k*≤*m*_ *N*_*k*_ = *N*. When *l* ≠ *k*, *N*_*l*_∩*N*_*k*_ = *⌀*. For *i* ∈ *N*, *k*(*i*) denotes the index of the union containing player *i*, so *k*(*i*) is defined by the relation *i* ∈ *N*_*k*(*i*)_. For *k* ∈ *M* and *S*⊆*N*_*k*_, the game ${\hat{v}}_{S}$ is defined by

(3) $$\begin{array}{c} {\hat{v}}_{S}\left(Q\right)=\{\begin{array}{cc} v\left(\underset{h\in Q}{⋃}N_{h}\right), & k\notin Q\\ v\left(\underset{h\in Q\setminus \left\{k\right\}}{⋃}N_{h}\cup S\right), & k\in Q, \end{array} \end{array}$$

where *Q*⊆*M*.

The game ${\overset{-}{v}}_{k}$ is defined by ${\overset{-}{v}}_{k}(S)=Sh_{k}({\hat{v}}_{S})$; then the Owen value of the player *i* ∈ *N* in the game ${\overset{-}{v}}_{k(i)}$ is $Ow_{i}(v,P)=Sh_{i}({\overset{-}{v}}_{k(i)})$.

### 3.2. The Critical Node Discovery Algorithm Based on Owen Value

The idea of the greedy algorithm is to find a node with the greatest influence during an iteration based on the diffusion model of social networks. In nature, the node with the maximum marginal contribution is chosen. Because the community structure is prevalent in the social networks [26], we, respectively, consider the community's influence on the information diffusion and every node's influence in the community. We take the nodes in the social network as the players in the cooperative game and information diffusion as coalition formulation. Thus, we can define an appropriate cooperative game for mapping the information diffusion in the social network and identify the critical nodes through the node's marginal contribution.

A network is a list of which pairs of players are linked to each other. The network structure is the key determinant of the level of productivity or utility to the society of players involved. A network game consists of a set of players and a value function. The value function assigns a real value to each possible network on all players. An allocation rule is a way to allocate the real value generated by a set of players and has to take into account the marginal value of a player. If we take the value function as the characteristic function, then network game can be seen as the cooperative game with transferable utility [27].

We define the cooperative game (*N*, *v*) where *N* is the set of nodes in the social network and the characteristic function *v* : 2^*N*^ → ℝ, 2^*N*^ is the set of all subsets of *N*. For each *S*⊆*N*, if all the nodes in *S* are initially activated, then *v*(*S*) represents the expected number of active nodes at the end of the diffusion process; that is, *v*(*S*) = *σ*(*S*).

So, we can use the Owen value to obtain the marginal contribution of every node. Because the Owen value can be seen as a two-step procedure in which the Shapely value is applied twice, we firstly compute a node's Shapely value.

Given a node *i* ∈ *N* and a subset *S*⊆*N* such that *i* ∉ *S*, the marginal contribution of node *i* is *v*(*S* ∪ {*i*}) − *v*(*S*), ∀*S*⊆*N*∖{*i*}. Consider the set of all possible permutations Ψ on *N*, let *ψ* ∈ Ψ, and define *S*_*i*_(*ψ*) to be the set of all nodes appearing before node *i* in the permutation *ψ*. So, the average marginal contribution of node *i* to the given coalitional game is

(4) $$\begin{array}{c} \frac{1}{n!}\sum_{\psi \in \Psi }\left[v\left(S_{i}\left(\psi \right)\cup \left\{i\right\}\right)-v\left(S_{i}\left(\psi \right)\right)\right]. \end{array}$$

Note that this method must work with *n*! permutations and its computational complexity is *O*((*n*/*e*)^*n*^) [28]. Therefore, we give the approximate method for computing Shapely value. Randomly generate a set Ψ_*t*_ with *t* permutations; let *ψ* ∈ Ψ_*t*_ and *ψ*(*i*) denotes the *i*th node in the permutation. The number of activated nodes after running the diffusion model when the node *ψ*(1) is activated is the contribution of *ψ*(1). Next, we consider the node *ψ*(2). If *ψ*(2) becomes active after *ψ*(1) is activated, then the contribution of *ψ*(2) is 0. Otherwise, the contribution of *ψ*(2) is the number of activated nodes by *ψ*(2). Therefore, we can get the contributions of *ψ*(3),…, *ψ*(*n*). For *ψ* ∈ Ψ_*t*_, repeat the above process *R* times. Then the average contribution of each node in the diffusion process can be calculated. We can obtain the top-k nodes sorted by the greatest influence and ensure that they are not adjacent to each other. See Algorithms 2 and 3.

According to the description in Section 3.1, we give the calculation method of the Owen value. Roger Guimerà et al. study node roles in the community according to within-module degree *z* and participation coefficient *P*, which are divided into seven categories, Role = {*R*1, *R*2, *R*3, *R*4, *R*5, *R*6, *R*7} [29]. We consider two types of the roles: nonhub connector node (*z* < 2.5 and 0.62 < *P* ≤ 0.80, *R*3) and connector hub (*z* ≥ 2.5 and 0.30 < *P* ≤ 0.75, *R*6). Most of the nodes which belong to these two roles connect to other communities.

We use the CNM algorithm [30] to divide the network *G* = (*V*, *E*) into *l* communities *C* = {*C*_1_, *C*_2_,…, *C*_*l*_ | *C*_*i*_ = (*V*_*i*_, *E*_*i*_), *i* = 1,…, *l*} and assign the role *rv*_*i*_^*h*^ ∈ Role for every node *v*_*i*_^*h*^ ∈ *V*_*i*_ in the community. Let Role′ = {*R*3, *R*6}, *i* = 1,…, *l*, *j* = 1,…, *l*,

(5) V′={vih,vjg ∣ ∃(vih,vjg),  vih∈Vi,  rvih∈Role′,  i≠j},E′={eih,(vih,vjg) ∣ eih=(viha,vihb)∈Ei,viha∈Vi, vihb∈Vi,  rvih∈Role′,  i≠j};

define *G*′ = (*V*′, *E*′) as community game network. So, we can obtain the Shapely value of every node in the network *G*′ and take the sum of the Shapely values of all nodes in the same community as the community's payoff. Then we treat a community as a separate network and calculate the Shapely value of every node in the community. The node's Owen value is assigned according to the normalized Shapely value of the node in the community and the community's payoff. So, we can get the *k* critical nodes by Algorithm 3.

### 3.3. The Computational Complexity

We consider the computational complexity for *k* − *CNP* based on Shapely value. In Algorithm 2, we compute the marginal contribution of every node and its complexity is *O*(*t*(*n* + *m*)*R*); the cost of ranking the contributions and selecting *k* nodes is *O*(*n*log⁡(*n*) + *kn*). Therefore, the overall running time of Shapely value-based algorithm is *O*(*t*(*n* + *m*)*R* + *n*log⁡(*n*) + *kn*). Because it is reasonable to assume that *n* < *m* for the real-world graphs and the Owen value can be seen as a two-step procedure in which the Shapely value is applied twice, the computational complexity of Owen value-based algorithm for *k* − *CNP* is *O*(*tmR*), where *t* is a polynomial in *n*.

## 4. Evaluation

We validate our method on two synthetic datasets and six real network datasets. All experiments are executed in the PC with 3.2 GHz CPU, 4 G memory, and Windows 7. The development tools are MATLAB 2009 and Microsoft Visual Studio 2010.

We compare our method (the Ov algorithm) with the Shapley value-based algorithm (the Sv algorithm), the greedy algorithm (the greedy algorithm), and the degree-heuristic algorithm (the degree algorithm). The greedy algorithm is as a benchmark for measuring other algorithms; the degree algorithm selects *k* nodes with the greatest degree from the network as the initial set. In order to obtain the accurate influence of every algorithm, we use the average number of the activated nodes after running ICM and LTM 10000 times for every initial set. In ICM, the propagation probability is set to 0.05; in LTM, the edge weight of a node is the reciprocal of the node's degree. The size of the initial set is, respectively, set from 1 to 20. The *t* in the Ov and Sv algorithms is shown in Table 1. In the experiments, we only discuss the case of using the ICM because we obtain the same conclusions for ICM and LTM.

### 4.1. Performance Comparison in Synthetic Networks

We consider the influence of community structure on the Ov algorithm.

We use BA model [31] and the forest fire model [32] to generate two synthetic datasets with 5000 nodes. The BA model takes the power-law distribution as two important features: growth and preferential attachment. The model can generate a scale-free network whose power exponent is 3 and community structure is not obvious. The forest fire model can generate a network with degree power law, densification law, shrinking diameter, and obvious community structure.

**Figure 1:**
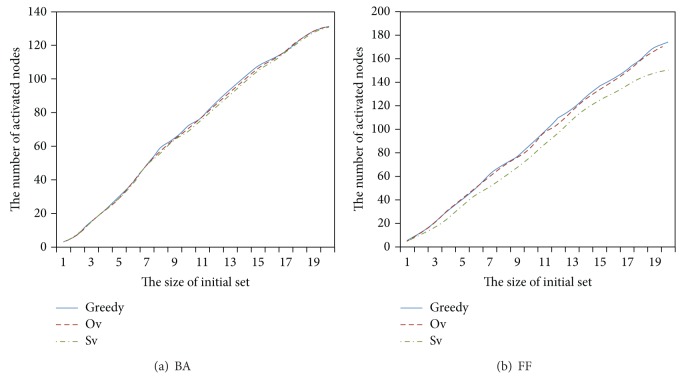
The influence of community structure on our method.

We, respectively, compute the Shapely value and Owen value of every node in the BA and FF datasets and obtain the initial set and the number of activated nodes after running the ICM. The process is repeated 100 times and the average number of the nodes activated by the initial set with different size is drawn in Figure 1. The Sv algorithm is almost the same as the Ov algorithm on BA dataset with unobvious community structure (Figure 1(a)). In contrast, the Ov algorithm is significantly better than the Sv algorithm on FF dataset with obvious community structure (Figure 1(b)). In Figure 1, the number of activated nodes by the initial set from the Ov algorithm is almost the same as the greedy algorithm.

### 4.2. Performance Comparison in Real Networks

The real datasets used by the paper include DBLP, Facebook, Enron, Youtube, AS, and PG, where the former four datasets have obvious community structure and the latter two datasets have not. The DBLP dataset [33] constructs a coauthor network with 143276 nodes and 359812 edges according to the papers published in the important conferences and journals of computer field from 1997 to 2006. The Facebook dataset [34] builds the friendship network of the New Orleans area with 60567 users and 583766 connections obtained from January 1, 2007, to December 31, 2008. The Enron dataset [35] is an E-mail network with 36692 nodes and 367662 edges. The YouTube dataset [36] consists of 35468 nodes and 261191 edges obtained from January 1, 2007, and January 15, 2007. The AS dataset [37] with 11456 nodes and 32759 edges is from the topology of autonomous systems on the Internet. The PG dataset [38] with 4941 nodes and 6594 edges is from the North American power grid.

**Figure 2:**
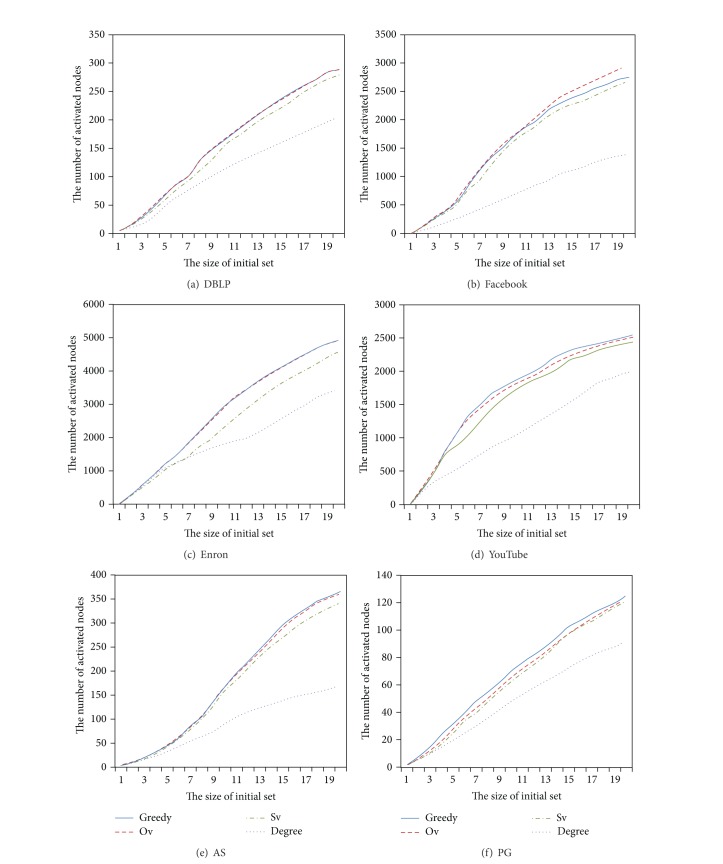
Performance analysis.

We, respectively, use the greedy, Ov, Sv, and degree algorithms to find out the initial sets from above six real datasets.  Figure 2 describes the number of the nodes activated by the initial set with different size based on ICM. From Figure 2, we see that whether on the networks with obvious community (Figures 2(a), 2(b), 2(c), and 2(d)) or on the ones with unobvious community (Figures 2(e) and 2(f)), the accuracy of the Ov algorithm is similar to the greedy algorithm, sometimes even better than it (Figure 2(b)). Compared with the Sv and degree algorithms, the Ov algorithm has a large advantage.

### 4.3. The Time Efficiency Comparison

We discuss our method's time efficiency.

**Figure 3:**
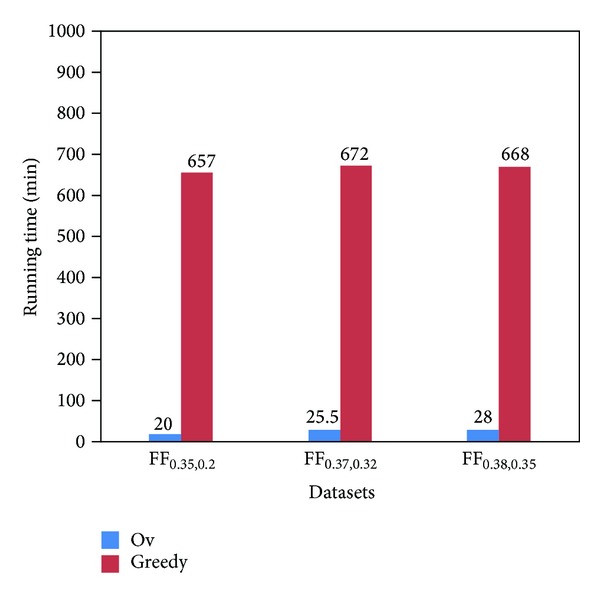
Time efficiency analysis.

We generate three datasets FF_0.35,0.2_ (sparse graph), FF_0.37,0.32_ (densifying graph), and FF_0.38,0.35_ (dense graph) with 1000 nodes using the FF model with three groups of parameters. Then we find out the top-20 critical nodes on these datasets by the Ov and greedy algorithms and plot the running times in Figure 3. We note that the Ov algorithm obtains a speedup of dozens of times over the greedy algorithm on these datasets.

### 4.4. An Example

We give an example of the Ov algorithm.

**Figure 4:**
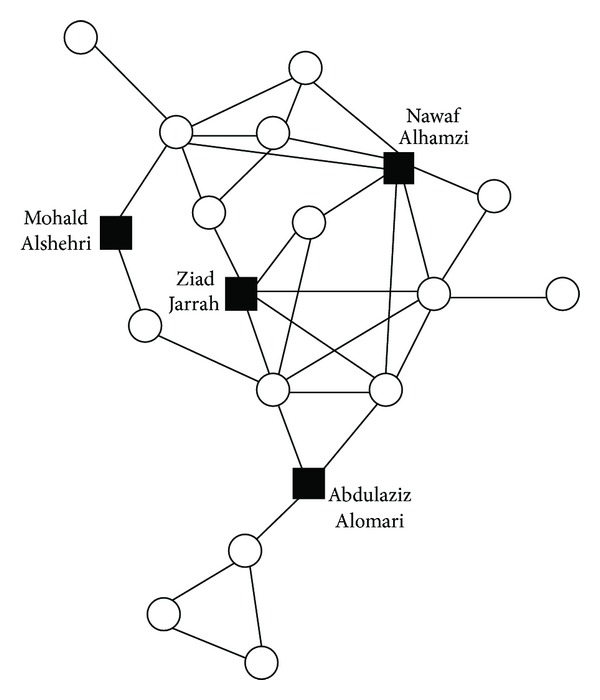
The terrorist network.

Krebs studied the terrorist network in the event of September 11, 2001.  Figure 4 and Table 2 show the trusted contacts among 19 hijackers [39]. Table 2 gives the names and airlines of these hijackers, and the squares in Figure 4 denote the critical nodes. We use the Ov algorithm to analyze the dataset and find out the sequence of critical nodes in which the first four people are Nawaf Alhamzi, Ziad Jarrah, Abdulaziz Alomari, and Mohald Alshehri. These four people are just on four different airlines which show that the Ov algorithm is effective to some extent.

## 5. Conclusions

For solving the CNP, this paper presents a method based on the Owen value from cooperative game, which considers the widespread community structure in social networks. We validate the proposed method on two synthetic datasets and the results show that our method is more suitable for the networks with community structure. Compared with other algorithms on six real datasets, our method is more effective. How to further improve the time efficiency of the Ov algorithm needs to be studied.

## Figures and Tables

**Algorithm 1:**
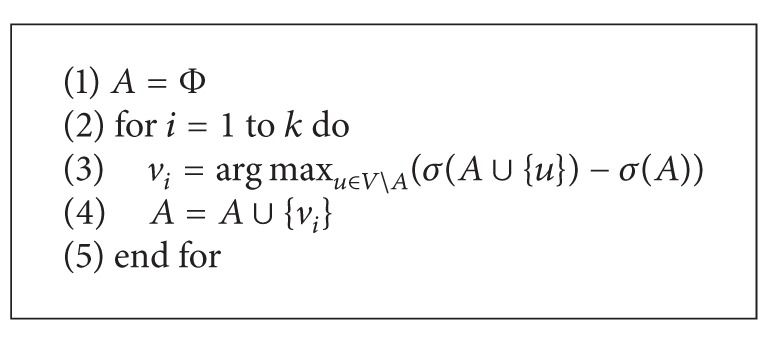
Greedy algorithm.

**Algorithm 2:**
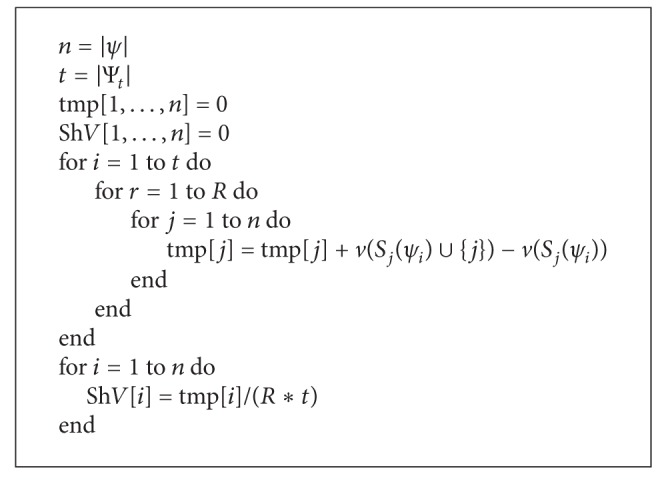
Shapely value(*v*).

**Algorithm 3:**
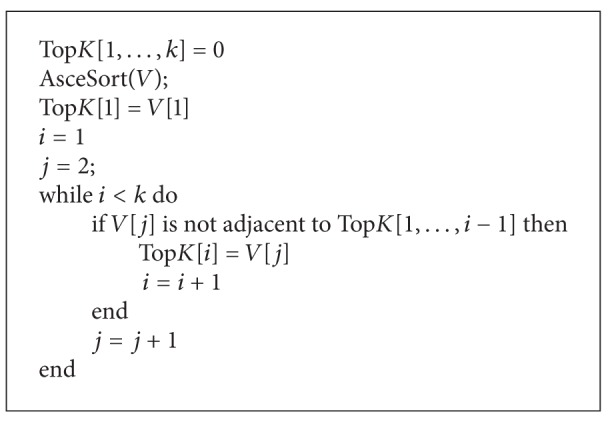
TOP_*K*(*V*).

**Table 1:** The *t* in the Ov and Sv algorithms for various datasets.

Datasets	*t*
BA	500
FF	500
DBLP	10000
Facebook	5000
Enron	4000
YouTube	3500
AS	1000
Power grid	500

**Table 2:** The hijackers on different airlines.

Airlines	Hijacker's name
American Airlines 11	Mohamed Atta, Waleed M. Alshehri, Wail Alshahri, Satam al-Suqami, *Abdulaziz Alomari *

American Airlines 77	Khalid al-Midhar, Majed Moqed, Salem Alhamzi, *Nawaf Alhamzi*, Hani Hanjour

United Airlines 93	*Ziad Jarrah*, Ahmed Alhaznawi, Ahmed Alnami, Saeed Alqhamdi

United Airlines 175	Marwan al-Shehhi, Fayez Ahmed, Ahmed Alqhamdi, Hamza Alghamdi, *Mohald Alshehri *
